# Visual Assessment of Movement Impairment During Walking in Subjects With Tibiofemoral Rotation Syndrome: A Concurrent Validity Study

**DOI:** 10.1016/j.jcm.2025.08.007

**Published:** 2025-09-25

**Authors:** Mehrnaz Kajbafvala, Ismail Ebrahimi Takamjani, Reza Salehi, Abbas Farjad Pezeshk, Fahimeh Firoozeh, Shahab Asgari, Abbas Tabatabaei

**Affiliations:** a Iranian Center of Excellence in Physiotherapy, Rehabilitation Research Center, Department of Physiotherapy, School of Rehabilitation Sciences, Iran University of Medical Sciences, Tehran, Iran; b Sport biomechanics, Faculty of Sport Sciences, University of Birjand, Birjand, Iran; c Mobility and Falls lab, Department of Physical Therapy, Rehabilitation Science, and Athletic Training, School of Health Professions, University of Kansas Medical Center, Kansas City, Kansas, USA

**Keywords:** Tibiofemoral, Visual Assessment, Motion Analysis

## Abstract

**Objective:**

This study aimed to assess the validity of visual assessment in diagnosing lower extremity impairments during level walking.

**Methods:**

Twenty-eight subjects (17 women, 11 men) with tibiofemoral rotation syndrome (TFRS) were examined using visual assessment as the index test and 3-dimensional (3D) motion analysis as the reference test. Both knee joint movements and the index and reference tests were evaluated simultaneously.

**Results:**

The sensitivity and specificity of visual assessment for detecting movement impairments were Se: 98% and Sp: 16.7%, respectively. The positive and negative predictive values were PPV: 81.5% and NPV: 50%, respectively. The positive and negative likelihood ratios were PLR: 1.18 and NLR: 0.12.

**Conclusion:**

The results demonstrate excellent validity of visual assessment for identifying movement impairments in subjects with TFRS during level walking. However, this test's validity for detecting the absence of movement impairments in subjects without TFRS was poor.

## Introduction

Lower extremity movement impairments may alter stresses at the knee and be associated with several pathologies.^1^, ^2^, ^3^ These impairments may manifest as any alterations in alignment or movement patterns during daily activities. Some evidence supports the relationship between lower extremity movement impairments and knee pain.^3^, ^4^, ^5^, ^6^ Dynamic knee valgus, a common impairment in the knee, could alter hip and knee kinematics in both frontal and transverse planes.^7^^,^^8^ This is characterized by excessive femoral adduction, internal femoral rotation, knee abduction, and external tibial rotation.^9^, ^10^, ^11^, ^12^ An excessive dynamic knee valgus may contribute to anterior cruciate ligament (ACL) tears^10^, ^11^, ^12^ or patellofemoral pain (PFP) during the landing or stance phase of gait.^8^

Identifying these alterations could improve a patient's symptoms, increase performance, and prevent further damage.^3^^,^^4^^,^^13^ Valid and practical approaches are needed to facilitate the assessment of movement impairments and consider the predictive factors of lower extremity injuries.^13^

The gold standard for assessing movement patterns is 3-dimensional (3D) motion analysis,^14^ which provides accurate 3D lower extremity measurement during functional tasks.^15^^,^^16^ Whatman et al.^17^ and Maclachlan et al.^18^ suggested that 3D motion analysis is a standard criterion for detailed kinematic evaluation. Lenezie et al. reported high within-day reliability for motion analysis.^16^ However, its application in the clinical setting is limited due to the high financial cost, time-consuming data collection, and demanding analysis.^19^

Visual assessment is the most clinically available and has higher accessibility for clinicians. It is commonly used in diagnosing, preventing, and treating musculoskeletal disorders in clinical practice.^17^ Clinicians use visual assessment in different ways to diagnose movement impairments. Sahrmann developed a standardized physical examination for lower extremity problems based on the classification of movement system impairment (MSI).^3^ The MSI system classifies patients into homogenous subgroups intending to increase the effectiveness of treatment.^20^, ^21^, ^22^, ^23^ The findings of a visual examination lead to the assignment of the MSI-related diagnosis named regarding the alignment and movement that seem to be related to the patient's symptoms.^3^ However, if a movement pattern is assessed based on visual observation, its validity must be distinguished compared to more quantitative movement analysis. Agreement between visual observation and motion analysis may help identify risk factors for lower extremity disorders and guide clinicians in selecting appropriate treatment strategies.

Tibiofemoral rotation syndrome (TFRS) is a category of MSI classification characterized by knee pain associated with impaired rotation of the tibiofemoral joint.^3^ Excessive rotation between the tibia and femur can be seen during directional testing, movement, and functional activity.^3^ Pain is often associated with activities that involve rotation between the tibia and femur, including weight-bearing activities such as walking and climbing stairs or non-weight-bearing activities such as sitting. In previous studies that examined the reliability and construct validity of the MSI classification in patients with knee pain, the proposed classification is known as reliable and valid. The tibiofemoral rotation syndrome was also recognized as a valid category.^24^^,^^25^

It is suggested that activities involving the whole body, such as walking, are more desirable than traditional measurements, like the range of motion, muscle strength, power, flexibility, and coordination, for evaluating movement patterns.^19^ Measuring movement using routine clinical tests is not recommended, as visual observation of functional activities, like walking, can assess all of these parameters at the same time.^17^^,^^26^^,^^27^

The validity of observational evaluation for therapists is essential in determining an accurate diagnosis and appropriate intervention. Therefore, it is necessary to compare this method with objective measurement tools and report its validity. The purpose of this study was to assess the concurrent validity of visual assessment and 3D motion analysis of walking in patients with TFRS. The study aimed to determine the correlation between visual assessment of lower extremity impairments during walking and the concurrent findings from 3D motion analysis, considered the gold standard for determining hip and knee alignment and movement patterns.

## Methods

### Participants

Adults with knee pain diagnosed with TFRS were recruited as a convenience sample from the Iran University of Medical Sciences, Tehran, Iran, between April and August 2022 through advertisements. Among the 44 patients who visited our unit during the study period, 28 patients (17 women, 11 men, age: 36.7 (15.4) years, height: 167.5 (7.2) cm, and BMI: 24.4 (5.2) kg/m²) met the inclusion criteria and were eligible for the study. Demographic characteristics, including age, gender, height, body mass index (BMI), visual analog scale (VAS- measures pain intensity, in which patients rate pain intensity from 0 [no pain] to 10 points [worst imaginable pain]), pain duration, and sport activity were provided by the examiner through demographic questionnaire. Inclusion criteria for the study were: (1) age between 18 and 65 years, and (2) sudden or gradual pain at the knee complex or surrounding tissues. Participants were excluded if they had any of the following: structural deformities of the spine or lower extremities used an assistive device, had a history of knee surgery within the last 3 months, had undergone more than 1 surgical operation on the knee, had a history of ligamentous injury diagnosis, had diabetes or were pregnant, had constant severe pain, were taking analgesic and anti-inflammatory drugs at the time of the study, showed signs of lumbosacral radiculopathy, neuromuscular disorders, rheumatoid arthritis (RA), or cardiopulmonary disease. All participants provided informed consent regarding the possible risks and benefits of the study. The study was approved by the Ethical Committee of the Iran University of Medical Sciences.

### Procedures

All tests were conducted in a biomechanics laboratory of Iran University of Medical Sciences during a single session. One of the investigators (MK) performed a visual assessment during level walking. This investigator was a physiotherapist with over 10 years of clinical experience in managing patients with musculoskeletal disorders based on the movement system impairment classification (MSI) approach and was also blind to the symptomatic side (see 2 supplementary video files). The second investigator (FF) recorded the kinematics of the lower extremity by 3D motion analysis simultaneously.

#### Walking kinematic analysis (3D motion analysis)

**Fig. 1:**
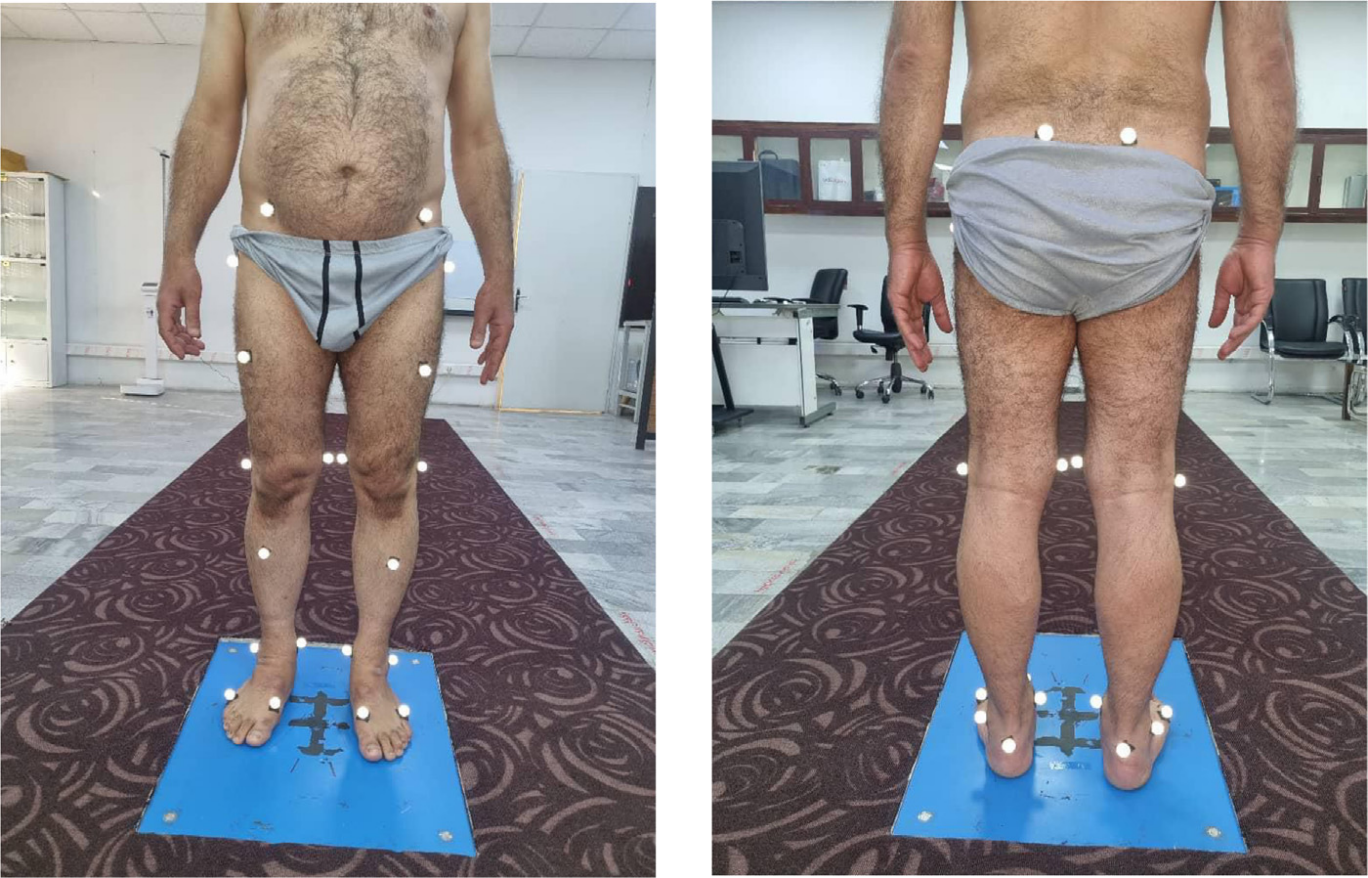
Alt Text: The location of retroreflective markers for 3D motion analysis.

**Fig. 2:**
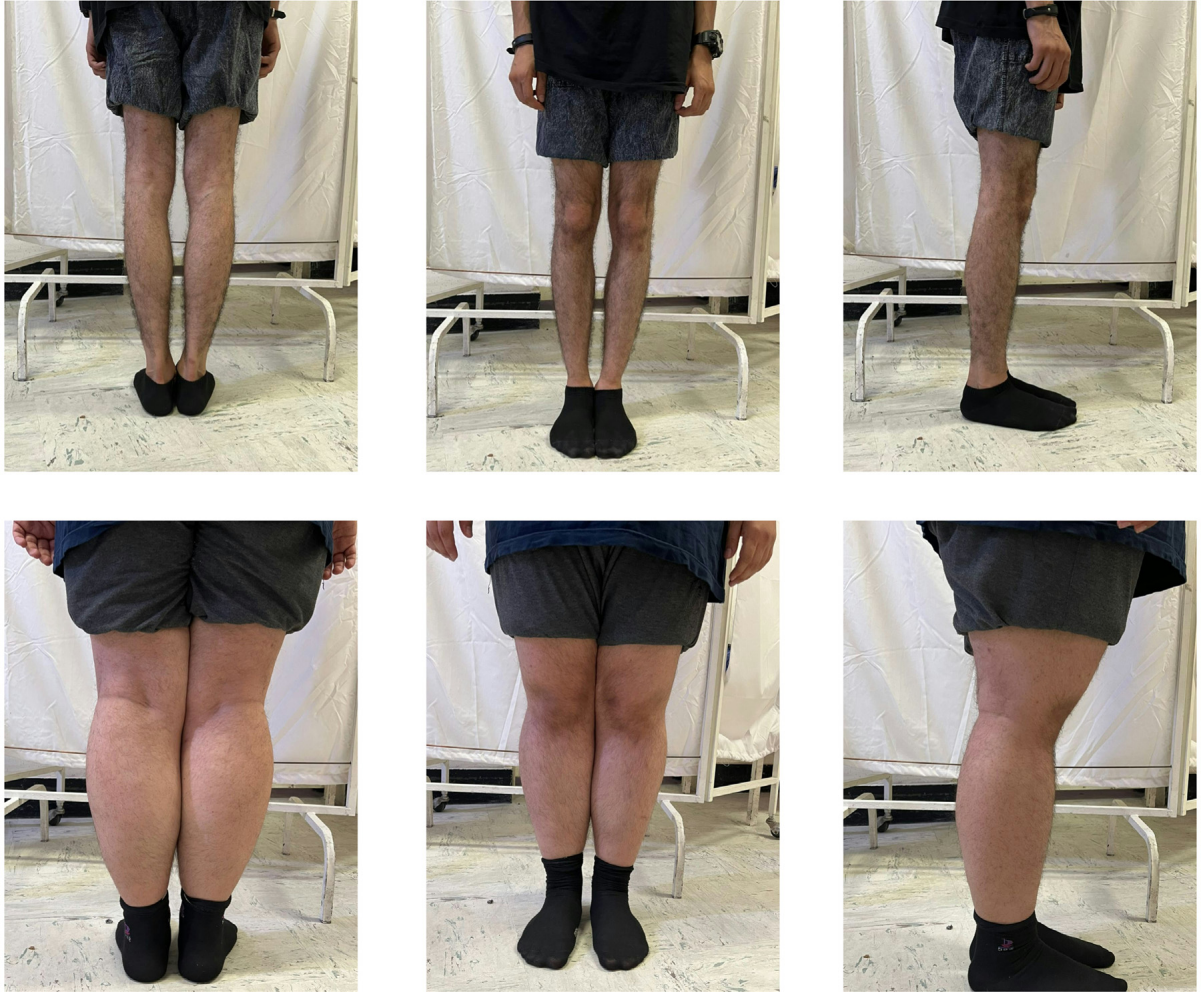
Tibiofemoral rotation with the valgus syndrome (lower row) and tibiofemoral rotation with the varus syndrome (upper row). Fig. 2 Alt Text: Images located in the lower row shows 3 views of a subject with Tibiofemoral rotation with the valgus syndrome, and the upper row images show 3 views of a subject with tibiofemoral rotation with the varus syndrome.

The kinematics of walking were assessed using a 3D motion analysis system (Qualisys, Gothenburg, Sweden) with 6 cameras (Oqus- 300) operating at a sampling rate of 100 Hz. The investigator attached 24 retroreflective passive spherical markers (21 mm in diameter) to specific anatomical landmarks in all participants, following modified plug-in-gait models (Fig. 1). The marker attachment method has excellent reliability in spatial-temporal and kinematic gait indices.^28^ An 8-meter space with a smooth surface covered by 6 motion cameras was used to monitor the markers and gait kinematics to assess the walking (Fig. 2). A relaxed standing calibration trial was performed before the walking test to calibrate the markers. Some practical walking trials were then conducted to familiarize all participants with the markers and environment. Participants subsequently walked barefoot in an 8-m walkway at their preferred, comfortable, and self-selected walking speed.^29^ One gait cycle was accepted for assessment based on 2 tests, including observational and 3D motion analysis. The gait cycle was defined as an interval between 2 successive heel strikes based on the lowest vertical position of the heel marker.^29^

#### Visual assessment

Before visual assessment during walking, a physical examination was performed. This examination included symptoms and signs data forms used to record findings.^24^ The symptom data form included the patient's responses to various test positions or movements. The sign data form consisted of the alignments and movement patterns that the patient considered, and the examiner judged to assess movement impairments. Excellent inter-rater reliability for the symptom items (kappa coefficient ≥ 0.75) and poor to excellent inter-rater reliability for the sign items (kappa coefficient = 0.18-1.00) of the knee MSI classification have been reported previously.^24^ For the visual assessment, the investigator positioned behind the participants at the start of the test and visually rated lower extremity movement impairment (maximum hip internal rotation) during 1 gait cycle regarding the MSI classification system.

### Study Design

The study had a cross-sectional design.

#### Sample Size

The sample size was calculated based on the potential specificity of 85%. As shown in the table of Buderer's study, the approximate range for the sample size was determined to be between 39 and 54 assessments with a power of 80% and alpha equal to 0.05. A sample size of 56, including 28 patients (56 knees) with tibiofemoral rotation syndrome, was determined for the sample size to avoid underestimation.

### Statistical and Data Analysis

Prior to statistical analysis, all variables were assessed for normal distribution using the Shapiro-Wilk test visually. Data analysis was performed using the statistical package STATA 13.0 (StataCorp, College Station, TX, USA).

Gait kinematic parameters related to TFRS were extracted from motion capturing, as shown in Table 2. Only the maximum hip internal rotation was used as a standard reference test to determine the rotation syndrome. The 3D motion analysis findings were converted into a yes/no qualitative variable for maximum hip joint internal rotation, which was then entered into the analysis. Due to the lack of a clear cut-off for converting kinematics variables related to hip joint qualitative variables, the present study used reported maximum hip internal rotation (mean+SD), which in the past study as the cut-off point to judge extreme hip internal rotation during the gait based. Therefore, movement impairment was assumed if a subject's maximum hip internal rotation was greater than 6.2°.^30^ A 2 × 2 table was used to provide descriptive validity aspects, with the index test as an independent variable in the columns and the reference test as a dependent variable in the rows.^31^

The "diag+" command and the "diagti TPFNFPTN" command were used to calculate and report validity parameters, including sensitivity, specificity, positive predictive value, negative predictive value, likelihood ratio (+), likelihood ratio (-), and prevalence, after verifying the assumptions. The corresponding 95% confidence intervals were calculated and reported using the Woolf method.^31^ Sensitivity as a tool validity parameter is interpreted with values between 87.5% and 100% as excellent, 75% to 87.5% good, 62.5% to 75% acceptable, and below 62.5% weak.^32^

## Results

The clinical characteristics of the subjects are shown in Table 1. The descriptive data related to lower extremity kinematics are presented in Table 2. In contrast, the descriptive and analytical statistics related to visual assessment of movement impairment are reported in Table 3, Table 4, respectively. The results showed that the sensitivity and specificity of observational evaluation of hip rotation were good to excellent (sensitivity: 98%, specificity: 16.7%) in subjects with the tibiofemoral rotational syndrome. The findings also revealed a positive predictive value (PPV) of 81.5%, negative predictive value (NPV) of 50%, positive likelihood ratio (PLR) of 1.18, and negative likelihood ratio (NLR) of 0.12.

**Table 1:** Baseline Clinical Characteristics

Clinical characteristics	Subject with TFRS (n=28) Mean (SD)
VAS (1w), 0-10	4.03 (2.14)
VAS (present), 0-10	1.92 (2.16)
PD (month)	3.8 (1.1)
With Sport Activity, (%)	67.86

PD, Pain Duration; VAS, visual analog scale (1 week before the test and during the test); TFRS, Tibiofemoral rotation syndrome.

**Table 2:** Kinematics Variables for Subject With Tibiofemoral Rotation Syndrome During Level Walking (n = 28)

Variable	Right^a^	Left^a^
Hip joint kinematics^b^		
Max internal rotation	7.1 (8.3)	7.9 (9.1)
Max external rotation	4.7 (9.8)	5.2 (8.8)
Time to max internal rotation^c^	49.2 (31.8)	42.5 (33.3)
Time to max external rotation^c^	64.8 (23.5)	67.9 (24.7)
Knee joint kinematics^b^		
Max abduction (Valgus)	5.1 (5.6)	48.9 (31)
Max adduction (Varus)	64.2 (24.7)	64.7 (19.9)
Time to max abduction^c^	3.6 (5.6)	3.9 (4.2)
Time to max adduction^c^	59.7 (30.7)	63.3 (29.9)

a Side of lower extremity.

b data are presented as mean (confidence interval).

c Percentage of gait cycle.

**Table 3:** Table 2 × 2 related to Observational Assessment of Movement Impairment in Subjects With Tibiofemoral Rotation Syndrome (56 knees)

	Motion analysis	
Observational assessment	1^a^	1^b^
	5^c^	49^d^

a True Negative.

b False Negative.

c False Positive.

d True Positive.

**Table 4:** Validity Values related to observational Assessment of movement impairment in patients with Tibiofemoral Rotation Syndrome

Validity parameters	Percentage	95% Confidence Interval
Sensitivity	98%	89.4%-99.9%
Specificity	16.7%	0.4%-64.1%
Positive Predictive Ratio	81.5%	68.6%-90.7%
Negative Predictive Ratio	50%	1.2%-98.7%
Likelihood Value (+)	1.18	0.01-1.7
Likelihood Value (-)	0.12	0.01-3.6
Prevalence	89%	78%-96%

## Discussion

The present study aimed to investigate the validity of observational assessment in diagnosing abnormal hip internal rotation caused by knee movement impairments. The findings indicated that the accuracy of the observational evaluation was excellent in this area. However, poor specificity was observed in different parameters of validity. In positive and negative tests, positive and negative predictive ratios (PPV and NPV) are more valuable than sensitivity and specificity.^31^ In this study, PPV demonstrated to what extent the movement impairment was present in knee rotational syndrome patients if the observation test was positive. Additionally, NPV indicated the probability of the absence of movement impairment in knee rotational syndrome patients if the observation test was negative. Due to the small sample size of this study and the high dependence of predictive ratios on prevalence, generalizing these parameters to the general population is limited. Therefore, sensitivity and specificity, which are not correlated to prevalence, are more important in explaining the present study. The sensitivity of the test demonstrated the portion of positive observational tests in subjects with approved movement impairment (confirmed by reference test or 3D motion analysis),^31^ and the findings showed that it was excellent. The sensitivity of a test reflects its contribution to separating affected individuals from the general population.^31^

Specificity represents the proportion of negative tests in the observational assessment of movement impairments in subjects with confirmed movement impairment (validated by a reference test or 3D motion analysis). In other words, the specificity of a test indicates its ability to distinguish healthy individuals from the general population. Unfortunately, in the current study, the specificity was poor, likely due to the limited number of patients without observable movement impairments (N = 1). Knee rotational syndrome, or tibiofemoral rotation syndrome (TFRVal or TFRVar) (Fig. 2), is among the most common knee joint movement impairments. Interestingly, TFRVal is more prevalent in females than males, a trend supported by existing literature.^3^

In the current study, the positive and negative likelihood ratios indicate the added value of observational assessment in diagnosing movement impairments. Generally, the closer these parameters are to 1, the less value the test adds to our information. However, the further they are from 1, the more informative they become. These parameters are not influenced by prevalence and can provide more precise interpretations in clinical settings. Based on the reported results in this study, the positive likelihood ratio had a better value than the negative likelihood ratio. In other words, a positive result in detecting a movement impairment was more accurate than a negative result.

The results of this study show that the visual assessment method is a valid measure for diagnosing knee rotational syndrome. The investigators developed this method based on the assumption that excessive rotation between the tibia and femur could be visually detected during tests of alignment, movement, and functional activity.^3^ The findings of this study support this assumption and demonstrate that the visual assessment method is a valid measure for assessing lower extremity movement impairments.

## Limitations

First, the subjects were recruited through university advertisements, and thus the sample may not be representative of all patients with knee complaints. Additionally, most subjects experienced mild to moderate pain, which may limit the generalizability of the results to patients with severe pain. A major limitation of the study is the small sample size, which led to limited numbers of patients without observational detection of movement impairments, thus reducing the ability to generalize the findings to the general population. Furthermore, the assumption of the lowest specificity was made for calculating the sample size due to the lack of data in previous literature. Finally, the validity of the visual assessment method was evaluated by an investigator with over ten years of clinical experience in managing patients with musculoskeletal disorders using the MSI approach. We recommend that future studies include novice examiners who have completed a short training course to repeat this validity study.

The findings of this study are clinically relevant as lower extremity movement impairments have been associated with increased transverse and frontal-plane hip movements (hip internal rotation/ hip adduction), frontal-plane knee movements (knee abduction/ adduction), and tibia external rotation.^8^^,^^9^, ^10^, ^11^, ^12^^,^^33^ These alterations have been linked to several conditions, including patellofemoral pain, ACL rupture, patellar instability, and iliotibial band syndrome, which could increase the risk of re-injury. Identifying these impairments is crucial for clinicians to prevent, diagnose, and treat patients with lower extremity movement impairments. Various methods for evaluating lower extremity movement impairments have been identified, including full-leg radiographs, knee radiographs, 3-D motion analysis systems, visual assessment, goniometers, inclinometers, or calipers. The visual assessment offers advantages over the 3D motion analysis system, considered the gold standard approach. It is more comfortable, accessible, and less costly for the patient, researcher, clinician, and/or healthcare system, does not expose the patient to radiation, and saves time.^18^^,^^34^ The results of this study apply to the visual assessment performed by a physiotherapist with sufficient experience in classifying lower extremity movement impairments based on visual assessment. These methods may enhance assessment by clinicians and researchers in patients with movement impairments of the knee.

## Conclusion

This study demonstrated that visual assessment was a valid measure for determining knee movement impairments during level walking. However, this test's validity for detecting the absence of movement impairments in subjects without TFRS was poor.

## Human Subjects and Animals

All participants signed an informed consent regarding possible risks and benefits of the study. The study was approved by the Ethical Committee of the Iran University of Medical Sciences.

## Funding sources and conflicts of interest

No funding sources or conflicts of interest were reported for this study.

## Contributorship Information

Concept development (provided idea for the research): M.K. Design (planned the methods to generate the results): M.K., A.T., A.F.P. Supervision (provided oversight, responsible for organization and implementation, writing of the manuscript): M.K., A.T. Data collection/processing (responsible for experiments, patient management, organization, or reporting data): M.K., F.F., S.H.A. Analysis/interpretation (responsible for statistical analysis, evaluation, and presentation of the results): A.F.P., M.K., A.T. Literature search (performed the literature search): MK, AT. Writing (responsible for writing a substantive part of the manuscript): M.K., A.T. Critical review (revised manuscript for intellectual content, this does not relate to spelling and grammar checking): R.S., I.E.T.

**Practical Applications**

• Validity of the visual assessment for movement impairments in subjects who have TFRS was excellent.
• There was poor validity regarding detecting the no movement impairments in the subjects without TFRS.
• Clinicians may consider use of visual assessment to assess movement impairment.

Alt-text: Unlabelled box

## Appendix Supplementary materials

Image, video 1

Image, video 2

## Footnotes

Supplementary material associated with this article can be found in the online version at doi:10.1016/j.jcm.2025.08.007.
